# Carbohydrate Starvation in Typhoid Fever

**Published:** 1896-02-22

**Authors:** Eric Pritchard

**Affiliations:** M.A., M.B., B.Ch.(Oxon.).


### Carbohydrate Starvation in Typhoid Fever.

By Eric Pritchard, M.A., M.B., B.Ch.(Oxon.).

In reviewing the various recommendations for the dietary of typhoid patients, as expressed in our best-known text-books on medicine, one cannot help observing the almost uniform agreement that 2 to 3 pints of milk, in addition to 1 to 1½ pints of beef-tea, is the diet that should be persevered with during the fever stage, and that the addition of extra nitrogenous food, such as eggs, &c., is indicated as the fever subsides. Is this uniformity of opinion to be attributed to the undoubted adequacy of such a diet, both on theoretical grounds and from clinical experience, or is it because from some reason or other it has been accepted as the classical standard from which physicians hesitate to depart? Without questioning the value of milk as the staple diet for typhoid patients, as well on dietetic as on practical grounds, there seem to me to be reasons for believing that milk can be modified for this purpose with advantage, just as cow's milk can be modified with advantage (humanised) to meet the requirements of infant feeding. On the other hand, I can see no reasons for supplementing such a milk diet with addition of beef-tea or other prepared extract of meat. Many years ago, H. v. Hoesslin proved by experiment that milk was well adapted for the purpose of feeding enteric fever cases; indeed, a larger percentage of the milk was digested and utilised than was the case with any mixed diet tried in his experiments. Analysis of his figures showed that while the carbohydrates present in milk were practically absorbed in their entirety, and the proteids almost equally well utilised, the fats were but indifferently taken up by the lacteals, and appeared in considerable quantities in the fæces. Arguing from these results, is one justified in believing that the degree of absorbability of these various constituents is some indication of the proportions in which they should be combined in an adequate diet for typhoid patients? In fact, should not the carbohydrates be increased in quantity, and the proportion of fat decreased?

To deal first with the question of the fats, there seems no particular reason, on theoretical or practical grounds, why they should constitute as large a proportion of the whole diet as they would form if natural milk were the sole diet. In typhoid fever the destruction of fat in the tissues is relatively small as compared to the albuminous metabolism. Fats are indifferently digested and absorbed, and they are not required for the maintenance of the natural body heat; and, further than this, the appetite naturally rebels against oleaginous food of any description. For these reasons, if for no others, there seem some grounds for believing it desirable to reduce the ingestion of fats below the limit which is necessary or advisable in health. The normal quantity of fat in an adequate diet of health is about 70 grams; the total quantity of fat in three pints of milk is about 80 grams. According to this argument, the quantity of fat given to typhoid patients is unnecessarily great, and should be reduced below that contained in three pints of natural milk.

The question as to the quantity of proteid required in conditions of continued fever always has been, and still is, a controversial subject of much difficulty. Von Ziemssen sums up his views on this point in the following sentence: "I believe that in fever a larger proportion of albuminates is requisite than in health, and so much larger as the albumen metabolism is greater." There is a danger, however, in increasing the nitrogenous diet beyond certain limits, since in itself a too free nitrogenous diet may lead to the breakdown of the fixed nitrogenous elements of the tissues, which, in addition to that which naturally occurs in continued fever, such as typhoid, may assume dangerous proportions. The total quantity of proteid food should, therefore, be slightly in excess of that which is required in conditions of health. For this purpose 80 grams of proteid, the quantity contained in two litres or three pints of milk, is, perhaps, hardly sufficient. If it should be thought desirable to increase this quantity, an addition of 20 grams of albuminous food may be effected. Some easily-assimilable proteid should be used to make good this deficiency, such as fresh meat juice. The addition of beef-tea or ordinary commercial extracts of meat is hardly fitted for this purpose, since they contain, in addition to the small proteid constituents, large quantities of extractives, which have little or no nutritive value, and only help to aggravate the already impure condition of the blood, loaded, as it is, with the products of nitrogenous metabolism due to the febrile state of the patient. It may, however, be contended that beef-tea or similar preparations are given as stimulants rather than as foods. If this be the only reason, less injurious stimulants, such as alcohol, strychnine, &c., should be substituted.

We must now discuss the important question as to the quantity of carbodydrates necessary for typhoid cases. In England it is an almost universal custom to depend solely on the carbohydrates contained in milk, namely, lactose or sugar of milk. Taking the amount of milk per diem at three pints, this would give less than 100 grams of sugar. The normal quantity of carbohydrates required by a healthy adult is about 400 grams, in addition to about 80 grams of fat. If his fat is absorbed, as is the case in typhoid fever, a large allowance of carbohydrate is required. Should there be this enormous difference (four to one) between the condition of health and those of continued fever? I have been quite unable in reading the literature of the subject to find any adequate reason for this carbohydrate starvation. Even in acute and short-lived conditions of fever no arguments are forthcoming for restriction of carbohydrates. Why should such a course be adopted when the febrile condition may continue for six or seven weeks or more? It seems to me that the same foods which it is expedient to give in short and acute attacks of fever, as measles, for instance, should be physiologically applicable in cases of longer continuance—typhoid, for instance; and yet in the one case carbohydrates are given freely; in the other they are reduced almost to the point of starvation. There is, however, a practical difficulty in adopting the same method in both cases, and that is the abnormal and unhealthy condition of the bowel in typhoid (in addition to an impairment of the physiological processes of digestion) which conduces to alcoholic, lactic, butyric, and acetic fermentation as soon as carbohydrates are admitted to the bowel in any but the smallest quantities.

